# Structural and functional characterization of NanU, a novel high-affinity sialic acid-inducible binding protein of oral and gut-dwelling Bacteroidetes species

**DOI:** 10.1042/BJ20131415

**Published:** 2014-02-28

**Authors:** Chatchawal Phansopa, Sumita Roy, John B. Rafferty, C. W. Ian Douglas, Jagroop Pandhal, Phillip C. Wright, David J. Kelly, Graham P. Stafford

**Affiliations:** *School of Clinical Dentistry, University of Sheffield, Sheffield S10 2TA, U.K.; †Department of Molecular Biology and Biotechnology, University of Sheffield, Sheffield S10 2TN, U.K.; ‡Department of Chemical and Biological Engineering, University of Sheffield, Sheffield S1 3JD, U.K.

**Keywords:** *Bacteroides fragilis*, carbohydrate, nanOU, outer membrane, sialic acid, transport, BF, *Bacteroides fragilis*, FA, fastidious anaerobe, HRP, horseradish peroxidase, ManNAc, *N*-acetyl mannosamine, NAM, *N*-acetylmuramic acid, NanO, neuraminate outer membrane permease, NanU, extracellular neuraminate uptake protein, Neu5Ac, 5-*N*-acetylneuraminic acid, Neu5Ac2en, 2-deoxy-2,3-didehydro-Neu5Ac, Neu5Gc, *N*-glycolylneuraminic acid, PUL, polysaccharide utilization locus, TBDR, TonB-dependent receptor, TCA, trichloroacetic acid, TF, *Tannerella forsythia*, TPR, tetratricopeptide repeat

## Abstract

Many human-dwelling bacteria acquire sialic acid for growth or surface display. We identified previously a sialic acid utilization operon in *Tannerella forsythia* that includes a novel outer membrane sialic acid-transport system (NanOU), where NanO (neuraminate outer membrane permease) is a putative TonB-dependent receptor and NanU (extracellular neuraminate uptake protein) is a predicted SusD family protein. Using heterologous complementation of *nanOU* genes into an *Escherichia coli* strain devoid of outer membrane sialic acid permeases, we show that the *nanOU* system from the gut bacterium *Bacteroides fragilis* is functional and demonstrate its dependence on TonB for function. We also show that *nanU* is required for maximal function of the transport system and that it is expressed in a sialic acid-responsive manner. We also show its cellular localization to the outer membrane using fractionation and immunofluorescence experiments. Ligand-binding studies revealed high-affinity binding of sialic acid to NanU (*K*_d_ ~400 nM) from two Bacteroidetes species as well as binding of a range of sialic acid analogues. Determination of the crystal structure of NanU revealed a monomeric SusD-like structure containing a novel motif characterized by an extended kinked helix that might determine sugar-binding specificity. The results of the present study characterize the first bacterial extracellular sialic acid-binding protein and define a sialic acid-specific PUL (polysaccharide utilization locus).

## Abstract 2

We biochemically and structurally characterize a novel inducible surface-associated sialic acid-binding protein present in human-dwelling *Bacteroidetes* species that represents a novel type of sialic acid-specific polysaccharide utilization locus.

## INTRODUCTION

The Bacteroidetes are a phylum of Gram-negative anaerobic bacteria that often have intimate relationships with their human or animal hosts, make up a large proportion of the natural intestinal flora and are dominated by species such as *Bacteroides fragilis* and *Bacteroides thetaiotaomicron*. Others, such as *Tannerella forsythia*, are associated with oral infections such as periodontal disease [1]. However, *B. fragilis* is also a commonly isolated nosocomial pathogen [2] and is also associated with bacterial vaginosis and pre-term birth [3]. The intimate relationship of these bacteria with their hosts is characterized by several aspects of their biology that include the ability to manipulate host metabolism [4,5] and modulate the host immune response [6,7]. In addition, this group of bacteria is characterized by possessing a wide range of glycosidase activities targeted at acquiring carbohydrate moieties both from their environment, but also directly from host glycoproteins [8,9]. Once released, the oligo- or mono-saccharide sugars are often acquired by dedicated transport systems that allow them to traverse the inner and outer membranes of these Gram-negative bacteria [10].

One component of the host glycome targeted by these bacteria is a family of nonulosonic acids known as sialic acids. The most prominent member of this family in humans is the 5-acetylated version known as Neu5Ac (5-*N*-acetylneuraminic acid). This sugar is present as the terminal moiety of a range of human glycoproteins, such as TLRs (Toll-like receptors) [11], integrins [12], mucins [9] and the blood group antigens SLeX/SLeA (sialyl-Lewis A/sialyl-Lewis X) [13]. Most commonly, it is linked to underlying sugars via α-2,3 or α-2,6 glycosidic linkages that are targeted by secreted or cell-associated sialidase enzymes from several members of the Bacteroidetes and other important human commensals and pathogens [8,9]. Once released, Gram-negative bacteria must transport free sialic acid across both their inner and outer membranes for use in catabolic pathways, or for reprocessing on to their cell surface [10]. In Bacteroidetes, catabolism initiates when sialic acid is converted into ManNAc (*N*-acetyl mannosamine) and pyruvate, by the action of a neuraminate lyase [NanA (*N*-acetylneuraminate lyase)] before an epimerase [NanE (*N*-acetylmannosamine-6-phosphate 2-epimerase)] converts ManNAc into GlcNAc (*N*-acetylglucosamine), that is phosphorylated by the RokA kinase before being turned into fructose 6-phosphate and subsequently used in glycolysis or cell wall biosynthesis [14].

There are several well-studied bacterial sialic acid inner-membrane transport systems, such as the SiaPQR TRAP (tripartite ATP-independent periplasmic) transporter from *Haemophilus influenzae* [15], the sodium solute symporter STM1128 from *Salmonella enterica* serovar Typhimurium [16], and the NanT MFS (major facilitator superfamily) permeases that are present in *Escherichia coli* and several Bacteroidetes species including *T. forsythia* and *B. fragilis* [8]. However, much less is known about how sialic acid traverses the outer membrane. This was first studied in *E. coli*, where the non-specific porins OmpC (outer membrane protein C) and OmpF (outer membrane protein F), but also the sialic acid-specific porin NanC (*N*-acetylneuraminic acid outer membrane channel protein) allow sialic acid to traverse the outer membrane [17]. More recently, our laboratory identified a novel sialic acid utilization operon in the *T. forsythia* genome that contains a putative TonB-dependent sialic acid-specific outer membrane transporter which we named NanOU [18]. It comprises a predicted β-barrel protein of the TBDR (TonB-dependent receptor) family, NanO (neuraminate outer membrane permease), adjacent to a smaller protein, NanU (extracellular neuraminate uptake protein) that has homology with the SusD family of proteins. The SusD proteins are outer membrane-associated proteins that act to bind and sequester oligosaccharide substrates that are transported by cognate SusC TBDR proteins, homologues of NanO [19,20]. Although the SusCD system from *B. thetaiotaomicron* is the best studied, they are part of a larger group of polysaccharide transport systems known as a PULs (polysaccharide utilization loci), which typically comprise a TBDR, a surface-associated binding protein, and a glycosidase or other sugar-processing enzyme [20]. Owing to this homology and a conserved genetic co-localization with sialidases, sialic acid processing and catabolism genes in Bacteroidetes species, we postulated that NanOU is a novel PUL that targets the monosaccharide sialic acid after its release from sialo-glycans attached to host surface glycoproteins. In the present study, we present genetic, biochemical and structural data that elucidates the function of NanU as a high-affinity sialic acid-binding protein that works in concert with its cognate TBDR, NanO, in sialic acid transport.

## MATERIALS AND MATHODS

### Growth media

*T. forsythia* (A.T.C.C. number 43037) was routinely grown anaerobically (10% CO_2_, 10% H_2_ and 80% N_2_) at 37°C on FA (fastidious anaerobe; Lab M) agar plates supplemented with 5% (v/v) oxalated horse blood containing 10 μg/ml NAM (*N*-acetylmuramic acid) and 50 μg/ml gentamycin. Liquid cultures were grown in trypticase soy broth plus 0.4% yeast extract supplemented with 10 μg/ml NAM, 50 μg/ml gentamycin, 5 μg/ml haemin and 1 mg/ml menadione. *B. fragilis* (NCTC 9343; a gift from Professor Sheila Patrick, Queen's University Belfast, Belfast, U.K.) was cultured identically, but in the absence of NAM and gentamycin. *B. fragilis* was also cultured on a defined media agar [1.5% (w/v)] adapted from the method described by Varel and Bryant [21] with modifications to the base medium by the addition of 1 mg/ml haemin (in 20 mM NaOH), 10 mg/ml menadione (vitamin K_3_), 0.74% methionine and 0.167% cobalamin (vitamin B12) with 15 mM glucose or Neu5Ac added as carbon sources. *E. coli* strains were routinely grown at 37°C in LB medium or M9 minimal medium as described previously [18] with appropriate supplements and carbon sources as indicated. Selective antibiotics were added to the appropriate concentrations as indicated. The strains used in the present study are listed in Supplementary Table S1 (at http://www.biochemj.org/bj/458/bj4580499add.htm).

### Production of Δ*tonB* strain and complementation plasmids

To construct a MG1655 Δ*nanCnanR*(*amber*) Δ*ompR*::*Tn10*(*tet*) Δ*tonB*::*FRT-Km-FRT* and an MG1655 Δ*tonB*::*FRT-Km-FRT* strain, we introduced a *tonB*-deletion mutation into our previously constructed MG1655 Δ*nanCnanR*(*amber*) Δ*ompR*::*Tn10*(*tet*) triple mutant and wild-type MG1655 using the gene-disruption method described by Datsenko and Wanner [22]. Briefly, *tonB* mutagenesis PCR products carrying a kanamycin-resistance gene were amplified from pKD13 plasmid using the primers EC*tonB*-FRT-F and EC*tonB*-FRT-R (Supplementary Table S2 at http://www.biochemj.org/bj/458/bj4580499add.htm) and used to transform the Δ*nanCnanR*(*amber*) Δ*ompR*::*Tn10*(*tet*) or MG1655 strain carrying the pKD46 helper plasmid with selection on LB plates with 50 μg/ml kanamycin. After curing of pKD46, the correct insertion was verified by PCR and sequencing while the phenotype of the single Δ*tonB*::*FRT-KM-FRT* mutation was confirmed by its dependency on cobalamin for growth.

To perform complementation experiments, *nanOU* homologues from *B. fragilis* NCTC 9343 were inserted into the arabinose inducible pBAD18 [23] plasmid before transformation into the strains indicated. The open reading frames of BF-NanO (*B. fragilis* NanO; *BF1719*; GenBank® accession number CR626927.1, region 2005661–2008927), BF-NanU (*BF1720*; region 2008979–2010526), and BF-NanOU (*BF1719-BF1720*; region 2005661–2010526) with stop codons at the 3′-end and an *E. coli* ribosome binding site (from pET15b) at the 5′-end were PCR-amplified using Phusion high-fidelity DNA polymerase (New England Biolabs) and the primer pairs BFO-XbaI-F/BFO-HindIII-R, BFU-XbaI-F/BFU-SphI-R and BFO-XbaI-F/BFU-SphI respectively (Supplementary Table S2 at http://www.biochemj.org/bj/458/bj4580499add.htm). The amplicons were inserted into pBAD18 via either XbaI-HindIII or XbaI-SphI doubly digested vector and inserted where appropriate. Along with the pBAD18 empty vector, the sequence-verified (Core Genomic Facility, University of Sheffield, Sheffield, U.K.) cloned constructs, named pCP-cBFO, pCP-cBFU, pCP-cBFOU and pCP-cTFOU, were used in the transformation of electrocompetent *E. coli* MG1655 Δ*nanCnanR*(*amber*) Δ*ompR*::*Tn10*(*tet*) and Δ*nanCnanR*(*amber*) Δ*ompR*::*Tn10*(*tet*) Δ*tonB*::*FRT-Km-FRT* strains, with selection on LB medium with 50 μg/ml ampicillin. Parental, mutant and the generated single/double-complemented strains were pre-cultured at 37°C overnight in M9 liquid medium containing 15 mM glucose as the carbon source. Strains with the Δ*tonB*::*FRT-Km-FRT* mutation were additionally supplemented with 88 μM iron (provided as FeCl_3_·6H_2_O) and 0.005 mM cobalamin. Stationary-phase cells were washed with sterile M9 medium, and used to inoculate fresh M9 medium with 0.5, 1, 3, 6 or 15 mM carbon source (glucose, Sigma–Aldrich, or Neu5Ac, Carbosynth) to a final *A*_600_ of 0.05 without induction with arabinose. Minimal medium growth experiments were carried out at 37°C, with absorbance measured (at *A*_600_) over time. Results are means of these three experiments and significant differences assessed using Student's *t* test.

### Production of recombinant NanU

The entire ORF of BF-*nanU* (*BF1720*) was PCR-amplified using Phusion high-fidelity DNA polymerase (New England Biolabs) from the genomic DNA of *B. fragilis* NCTC 9343 using the primers BF*nanU*-NdeI-F and BF*nanU*-XhoI-R (Supplementary Table S2). The amplicon was then inserted into the NdeI and XhoI sites of pET21a(+) (Merck Millipore) and verified by sequencing (Core Genomic Facility, University of Sheffield). The coding region of TF-NanU (*T. forsythia* NanU; GenBank® accession number CP003191.1, region 2360849–2362417) was synthesized in an *E. coli* codon-optimized construct by Eurofins MWG such that changes in codon usage resulted in no changes in the primary amino acid sequence. The gene was subcloned from the holding plasmid pEX-A into pET21a(+) using NdeI and XhoI, and its sequence was confirmed by DNA sequencing. *E*. *coli* BL21λ(DE3) cells were transformed with plasmids and grown in either LB or M9 (glycerol) broth-cultured cells with induction (1 mM IPTG; Sigma–Aldrich) during the mid-exponential growth phase (*A*_600_=0.6) for 5 h at 37°C with agitation. Cells were harvested and resuspended in 25 mM sodium phosphate, pH 7.4, 0.5 M NaCl and 25 mM imidazole, disrupted using a French pressure cell (Thermo Scientific) and soluble fractions clarified by further centrifugation (10000 ***g*** for 30 min at 4°C). The C-terminally His_6_-tagged proteins were purified by Ni^2+^–Sepharose affinity chromatography and eluted with a 50–200 mM imidazole gradient on the ÄKTAprime plus system (GE Healthcare). The purified proteins were extensively dialysed against 25 mM sodium phosphate, pH 7.4, concentrated using a MWCO (molecular-mass cut-off) 10000 Vivaspin column (GE Healthcare) and protein concentrations were determined using the Pierce BCA protein assay kit (Thermo Scientific). The identities of recombinant BF-NanU (BF1720) and TF-NanU (TF0034) were confirmed by LC–MS/MS (ChELSI, University of Sheffield).

### Sialic acid-binding studies

The binding characteristics of purified NanU with candidate ligands were investigated by steady-state tryptophan fluorescence spectroscopy [BF-NanU (BF1720) and TF-NanU (TF0034) possess seven and eight tryptophan residues respectively]. Proteins were diluted in 25 mM sodium phosphate buffer, pH 7.4, to a final concentration of 0.5 μM and incubated at 25°C for 5 min before substrate titration. The quenching of intrinsic tryptophan fluorescence was measured at 25°C with constant stirring in a 104F-QS quartz fluorescence semi-micro cell (Hellma Analytics) using a Varian Cary Eclipse spectrofluorometer. The excitation and emission wavelengths were 295 nm (5 nm slit width) and 330 nm (10 nm slit width) respectively. Corrections for background fluorescence changes due to dilution effects with the stepwise addition of ligands were made by buffer titrations. Analysis and curve fitting of titration data were performed in Prism 5 (GraphPad Software), with the equilibrium dissociation constants (*K*_d_) for one site-specific binding calculated using eqn (1):

(1) $$Y=B_{\max}\times X/\left(K_{d}+X\right)$$

where *X* is the final concentration of ligand, *Y* is the percentage increase in protein quenching in relation to the protein-only fluorescence signal and *B*_max_ is the extrapolated maximum specific binding.

### Crystallization and data collection of BF-NanU

Purified BF1720 was concentrated to 6 mg/ml in 25 mM sodium phosphate, pH 7.4, and tested for crystallization at 3 mg/ml with a variety of commercial screens. Subsequent optimization resulted in the growth of X-ray diffracting crystals grown with 1 μl of protein and 1 μl of reservoir solution [0.2 M ammonium acetate and 20% (w/v) PEG 3350] after 14 days of incubation at 17°C. Crystals were mounted direct from the drop without the addition of cryoprotectant and flash-cooled in a nitrogen gas stream maintained at 100 K. Data were collected to 1.6 Å on station I04-1 at the Diamond Light Source (Harwell, U.K.), and processed using XDS [24]. Molecular replacement was performed using the program PHASER on these data, employing a model obtained from the PHYRE^2^ server [25] based upon a SusD superfamily protein from *Bacteroides vulgatus* (PDB code 3JQ0). The initial model was improved by application of the PIRATE and BUCCANEER programs from the CCP4 suite [26] coupled to manual intervention in COOT [27] interspersed with rounds of refinement using REFMAC [28]. A summary of the relevant data statistics is shown in Supplementary Table S3 (at http://www.biochemj.org/bj/458/bj4580499add.htm). Structural factors and co-ordinates have been deposited in the PDB under code 4L7T. All Figures were generated using PyMOL (http://www.pymol.org) and the topology diagram was prepared using CorelDraw X4 (Corel Corporation).

To ascertain whether both native and ligand-bound BF1720 were monomeric, 3 mg/ml of purified protein with and without pre-incubated Neu5Ac in 25 mM sodium phosphate, pH 7.4, at an equimolar concentration were sequentially applied to a HiLoad Superdex 200 PG gel-filtration column (GE Healthcare) at a flow rate of 1 ml/min. Apoferritin (443 kDa), β-amylase (200 kDa), alcohol dehydrogenase (150 kDa), BSA (66 kDa), ovalbumin (43 kDa), trypsin inhibitor (20 kDa), cytochrome *c* (12.3 kDa) and aprotinin (6.5 kDa) were used as molecular mass standards.

### Production of an anti-NanU antibody and the localization of NanU

To produce a polyclonal antibody specific against BF1720, purified protein was injected into rats and the animals were subsequently boosted twice with the same antigen (BioServ U.K.). The specificity of the antisera was confirmed in test blots on the lysates of *B. fragilis*, *E. coli*, *T. forsythia* and *Porphyromonas gingivalis*, with only the NanU band highlighted in its native species and no cross-reacting bands present in the other three species even at high antisera titres (results not shown).

### Preparation of cellular and secreted fractions from Bacteroidetes cultures

Whole-cell samples were normalized according to the absorbance, i.e. 1 ml at *A*_600_=1.0 was resuspended in 200 μl of Laemmli sample buffer and 10 μl subjected to SDS/PAGE (10% gel). Cells were fractionated in a method adapted from previous work [29]. *B. fragilis* NCTC 9343 and *T. forsythia* A.T.C.C. 43037, plate-grown in an anaerobic atmosphere for 1 and 5 days respectively, were resuspended in 25 mM sodium phosphate, pH 7.4, and 0.5 M NaCl, placed on ice and disrupted by sonication using a Soniprep 150 (MSE). Cell debris was removed by two rounds of centrifugation (5000 ***g*** for 30 min at 4°C), and fractions containing membrane proteins were pelleted (330000 ***g*** for 3 h at 4°C). The supernatants were carefully decanted and stored (−20°C) as cytoplasmic fractions. Membrane pellets were washed twice in 25 mM sodium phosphate, pH 7.4, and 0.5 M NaCl, resuspended in 1 ml of the same buffer and an equal volume of 2% (v/v) sodium *N*-lauroyl sarcosinate in phosphate buffer (pH 7.4, 0.5 M NaCl) added, followed by incubation at 37°C for 30 min. The suspensions were centrifuged (10000 ***g*** for 30 min at 4°C), and the supernatants containing the inner membrane fractions stored (−20°C). The outer membrane pellets were washed twice in the same phosphate buffer before resuspending in 500 μl of the same buffer and stored at −20°C. To obtain fractions containing secreted proteins, the same *B. fragilis* and *T. forsythia* strains were cultured in 20 ml of liquid broth for 1 and 5 days respectively. Cells were removed by centrifugation (10000 ***g*** for 30 min at 4°C) before the addition of 5 ml of TCA (trichloroacetic acid) to each clarified supernatant and incubation at 4°C for 15 min. Proteins were pelleted by centrifugation (10000 ***g*** for 30 min at 4°C) and the resulting pellets washed three times with 500 μl of ice-cold acetone. The pellets were dried on a 95°C heat block for 10 min before resuspension in 500 μl of Laemmli buffer. Cell fractions were resolved by denaturing SDS/PAGE (10% gel), transferred on to nitrocellulose membranes (Millipore), blocked overnight with TBS-T (TBS, pH 7.4, containing 0.05% Tween 20), 5% (w/v) skimmed milk and 3% (w/v) BSA. The membranes were then probed with BF1720 rat antiserum before incubation with HRP (horseradish peroxidase)-conjugated goat anti-(rat IgG) (Sigma–Aldrich). Proteins were visualized using a Pierce enhanced ECL HRP substrate (Thermo Scientific) before incubation with CL-XPosure X-ray film (Thermo Scientific) and development using the Compact X4 automatic film processor (Xograph Healthcare). To detect the presence of possible cytoplasmic protein contamination, Western blots were performed as described above, but using anti-(*E. coli* GroEL rabbit IgG) (Sigma) and anti-(rabbit HRP-conjugated goat IgG) (New England Biolabs) as the primary and secondary antibodies respectively.

### Immunofluorescence microscopy

Poly-L-lysine coverslips (BD BioCoat) were briefly immersed in a PBS suspension of *B. fragilis* cells at *A*_600_=0.1 plate-cultured for 16 h, following which the cells were fixed with 4% (v/v) paraformaldehyde in PBS (buffered to pH 7.0) for 30 min at 37°C, blocked with PBS containing 3% (w/v) BSA for 1 h and incubated with BF1720 rat antiserum (1:5000 dilution) for 2 h. The coverslips were incubated in the dark with PBS containing Alexa Fluor™ 488 goat anti-(rat IgG) (1:3000 dilution; Life Technologies) for 1 h, and mounted on to glass slides with ProLong Gold antifade reagent with DAPI (Life Technologies). Fluorescence was visualized under a Zeiss Axiovert 200 M microscope at ×100 magnification. Repeated washes of coverslips with PBS were carried out between steps, and sample preparations and visualization were carried out at 25°C unless stated otherwise.

## RESULTS

### The *nanOU* homologues from *B. fragilis* restored sialic acid-dependent growth to an *E. coli* Δ*nanCR*Δ*ompR* mutant

**Figure 1:**
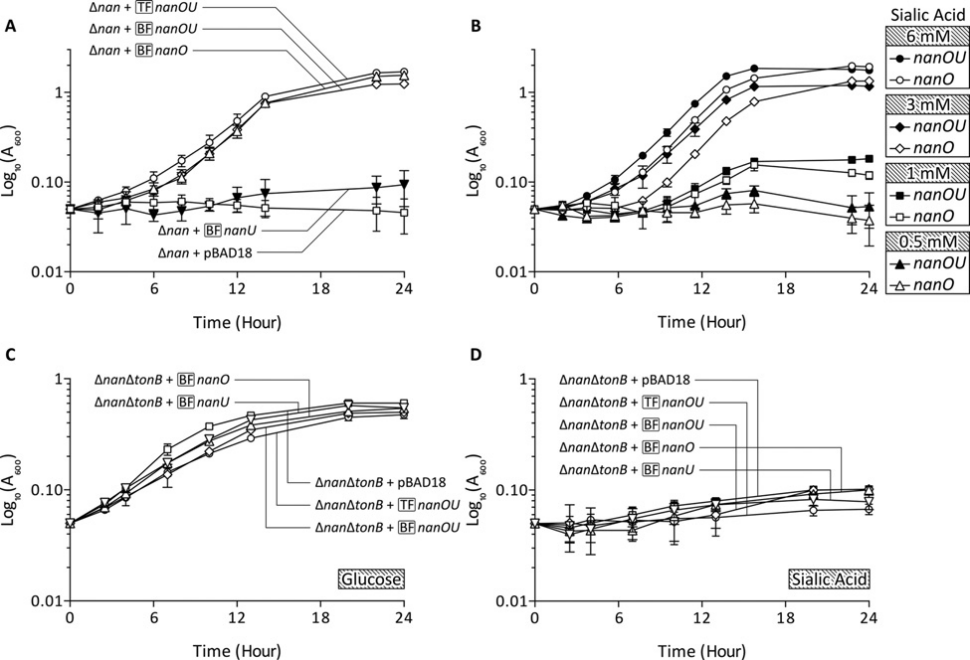
Ability of *nanOU* genes to support growth on sialic acid (**A**) Growth kinetics of *E. coli* MG1655 Δ*nanCnanR(amber)* Δ*ompR*::*Tn10*(*tet*) (Δ*nan*) mutant strains complemented *in trans* with the *nanOU* genes from *T. forsythia* (TF-*nanOU*, ○), *B. fragilis* (BF-*nanOU*, ∆) or the individual *B. fragilis nanO* (BF-*nanO*, ◇) or *nanU* (BF-nanU, ▼) expressed from pBAD18 (also included as a negative control, □) were monitored (*A*_600_) during culture at 37°C in M9 minimal media with 15 mM Neu5Ac. Results are means±S.D. from three separate biological replicates. (**B**) The Δ*nan* strain complemented with either *B. fragilis nanO* (BF-*nanO*, open symbols) or *nanOU* (BF-*nanOU*, close symbols) was incubated as above but with 6 (● and ○), 3 (◆ and ◇), 1 (■ and □) or 0.5 (▲ and ∆) mM Neu5Ac. Results are means±S.D. from three separate biological replicates. (**C** and **D**) The sialic acid transport and *tonB*-deletion mutant strains [Δ*nanCnanR*(*amber*) Δ*ompR*::*Tn10*(*tet*) Δ*tonB*::*FRT-Km-FRT*–Δ*nan*Δ*ton*] were complemented as in (**A**) and (**B**) in M9 medium with either 15 mM glucose (**C**) or Neu5Ac (**D**) as indicated and growth followed over time. Results are means±S.D.

As published previously by our group [8], several genomes of species in the Bacteroidetes group contain putative homologues of the *nanOU* putative sialic acid-transport genes, including those from *T. forsythia* and also several other human pathogens and commensal species. Among these, the genes with highest homology with *nanOU* from the oral pathogen *T. forsythia* are those from the Gram-negative anaerobe *B. fragilis* NCTC 1943, namely BF1719 (BF-*nanO*) and BF1720 (BF-*nanU*), which have 69.0% and 63.1% amino acid identity respectively. To investigate whether the putative *nanOU* genes from *B. fragilis* functioned in a similar manner to those from *T. forsythia* in sialic acid transport and to begin to establish whether they might form part of a novel family of sialic acid transporters, we inserted BF-*nanOU* into the pBAD18 expression plasmid and transferred them into an *E. coli* Δ*nanCnanR*(*amber*) Δ*ompR*::*Tn10*(*tet*) strain, which is devoid of all outer membrane sialic acid porins and is therefore unable to grow with sialic acid as a sole carbon and energy source [18]. We then assessed its ability to grow in M9 minimal medium with sialic acid as a sole carbon source. In the present study, no arabinose was added, as our previous experience is that complementation is achieved from these plasmids without induction [18]. In a similar manner to the TF-*nanOU* genes, the BF-*nanOU* genes were able to restore the sialic acid growth defect of this strain (Figure 1A). These data indicate that BF-*nanOU* not only encodes a functional sialic acid transport system, but also confirm that this may be a common sialic acid transport method in this group of important human-associated bacteria.

### NanU enhances NanO function

In order to determine the contribution of the individual *nanO* and *nanU* genes to sialic acid transport, we cloned the individual genes from *B. fragilis* NCTC 9343 into pBAD18, and tested their ability to restore the sialic acid growth defect of the Δ*nanCnanR*(*amber*) Δ*ompR*::*Tn10*(*tet*) strain, again without the addition of arabinose. As shown in Figure 1(A), when BF-*nanU* was provided *in trans* with 15 mM sialic acid in the growth medium, no restoration of the growth defect was observed, but when BF-*nanO* was provided in isolation, growth was restored to approximately wild-type levels. To probe further the role of NanU, we asked the question of whether at low Neu5Ac concentrations, *nanU* enhances *nanO* function. To address this question we repeated the experiment at a range of lower concentrations (0.5–6.0 mM) and as shown in Figure 1(B). We consistently observed a slight, but significant, increase in the length of the lag phase of the growth curve that is most prominent at 3 mM (i.e. the curve shifted to the right-hand side) as evidenced by lower *A*_600_ readings for the *nanO*-only strains at all time points between 6 and 22 h (*P*<0.05; one-tailed Student's *t* test), but also at 6 mM sialic acid. In addition, we observed a lower final growth yield at lower concentrations of sialic acid, i.e. when only 1 mM sialic acid was provided (*A*_600_=0.2 compared with 0.1) in comparison with the provision of BF-*nanOU* in concert. We consider it unlikely that these differences are due to differences in expression levels from these plasmids, since the promoter sequences are identical for the BF-*nanO* and BF-*nanOU* constructs and there was no difference in levels of inducer added (i.e. none). Thus the data indicate a dependence on both NanO and NanU for maximal activity in supplying sialic acid to internal catabolic pathways.

### NanOU activity requires a functional TonB–ExbB–ExbD system

As mentioned above, the NanOU system appears to be the first TonB-dependent system adapted for sialic acid transport. As a result, we wished to confirm the dependence of the observed sialic acid outer membrane transport by the NanOU system on the presence of a functional TonB–ExbB–ExbD complex. To achieve this, we first constructed an *E. coli* Δ*nanCnanR*(*amber*) Δ*ompR*::*Tn10*(*tet*) Δ*tonB*::*FRT-Km-FRT* (referred to as Δ*nan*Δ*tonB* in Figures 1C and 1D) strain by inserting a kanamycin-resistance cassette in place of *tonB* in the existing Δ*nanCnanR*(*amber*) Δ*ompR*::*Tn10*(*tet*) background [22]. When the growth of this strain on glucose was tested, it was unaffected by this mutation or the presence of the *nanOU* genes from *T. forsythia* or *B. fragilis* (Figure 1C). In contrast, there was no growth of this strain when sialic acid was provided as the sole carbon source, even in the presence of the *B. fragilis* or *T. forsythia nanOU* genes *in trans* (Figure 1D), indicating that the function of *nanOU* is TonB-dependent.

**Figure 2:**
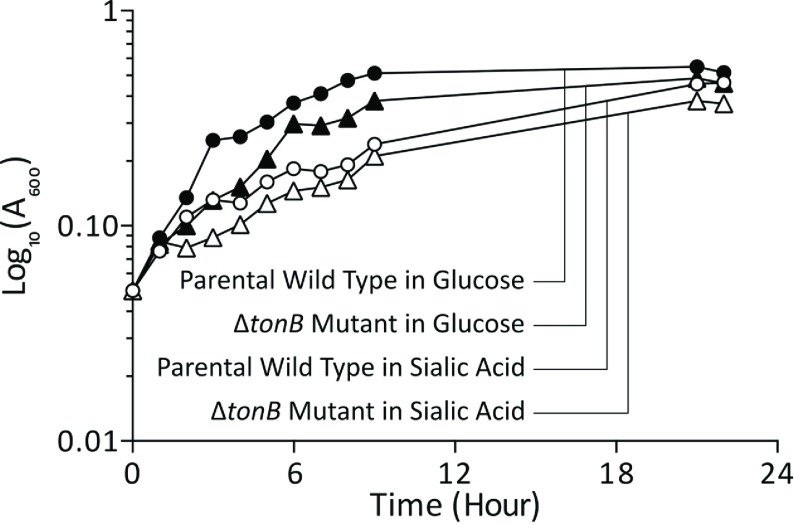
Role of *tonB* in a *nan* (transport)-positive strain The Δ*tonB* strain was grown in M9 medium containing 15 mM Neu5Ac and growth followed over time. Results are means±S.D. from three separate biological replicates.

To exclude the possibility that TonB may have a role in sialic acid uptake itself in *E. coli*, we constructed a Δ*tonB*::*FRT-Km-FRT* strain (using the same primer set) in the isogenic wild-type *E*. *coli* MG1655 background. As shown in Figure 2, both the wild-type and Δ*tonB* strains were able to grow well on both glucose and sialic acid as a carbon source, indicating that the function of *nanOU* is dependent on the presence of a functional TonB–ExbB–ExbD complex.

### NanU expression is altered by the presence of sialic acid

**Figure 3:**
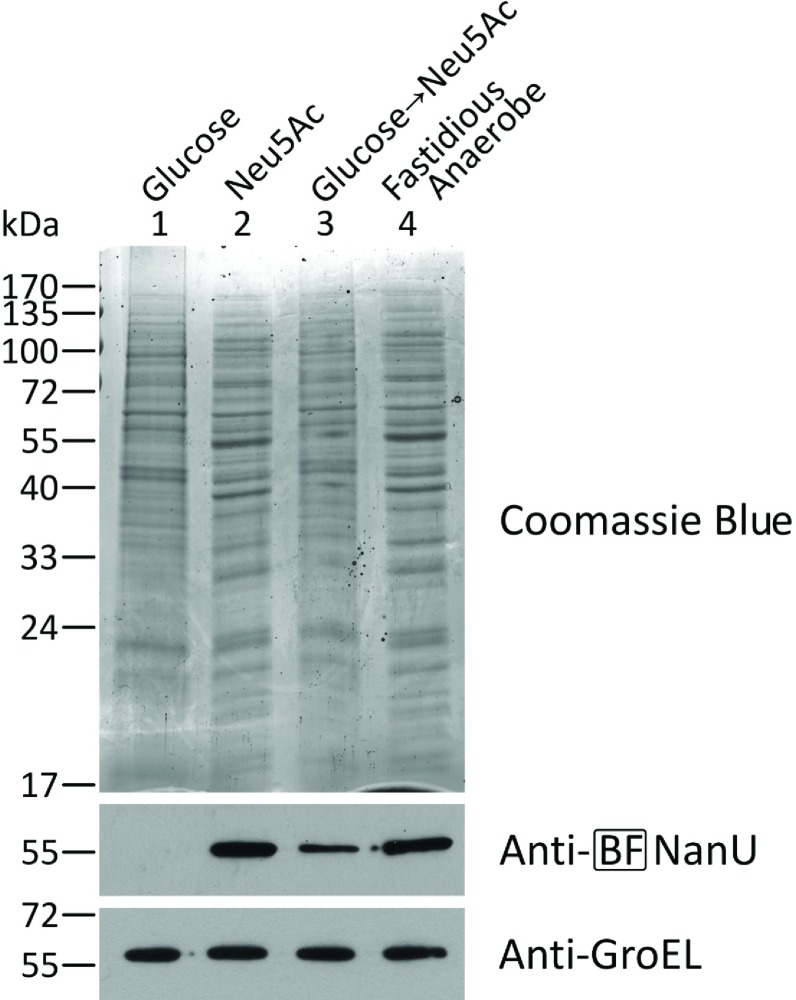
NanU expression in *B. fragilis* in response to different carbon sources *B. fragilis* NCTC 9343 cells cultured overnight on FA agar were subcultured on FA agar (16 h) (lane 4) or minimal medium agar supplemented with 15 mM glucose (lane 1), Neu5Ac (lane 2) or glucose (16 h) then Neu5Ac (16 h) (lane 3). Normalized amounts of proteins were stained with Coomassie Blue or probed with rat BF-NanU antiserum or rabbit anti-(*E. coli* GroEL) as described above. Molecular mass is given on the left-hand side in kDa.

To investigate the conditions under which NanU might be expressed, we examined its expression profile in whole cells grown in defined medium. We used our anti-NanU antibody as a probe in Western blot analysis (Figure 3) with cell mass normalized by cell number and blots probed with an anti-GroEL antibody as a loading control. When *B. fragilis* was grown on defined medium agar plates with glucose as the carbon source, we were unable to detect NanU in the whole-cell fractions. In contrast, when grown on the same defined medium containing sialic acid in place of glucose, expression was detected strongly. Similarly, when cells grown on glucose were transferred on to sialic acid-containing plates, we again observed an increase in NanU expression by sialic acid. In addition, we observe strong expression of NanU on rich FA blood agar plates, where the blood cells probably act as a source of sialic acid. Taken together, these data indicate strong sialic-dependent NanU expression in *B. fragilis.*

### NanU is an outer membrane-associated protein

**Figure 4:**
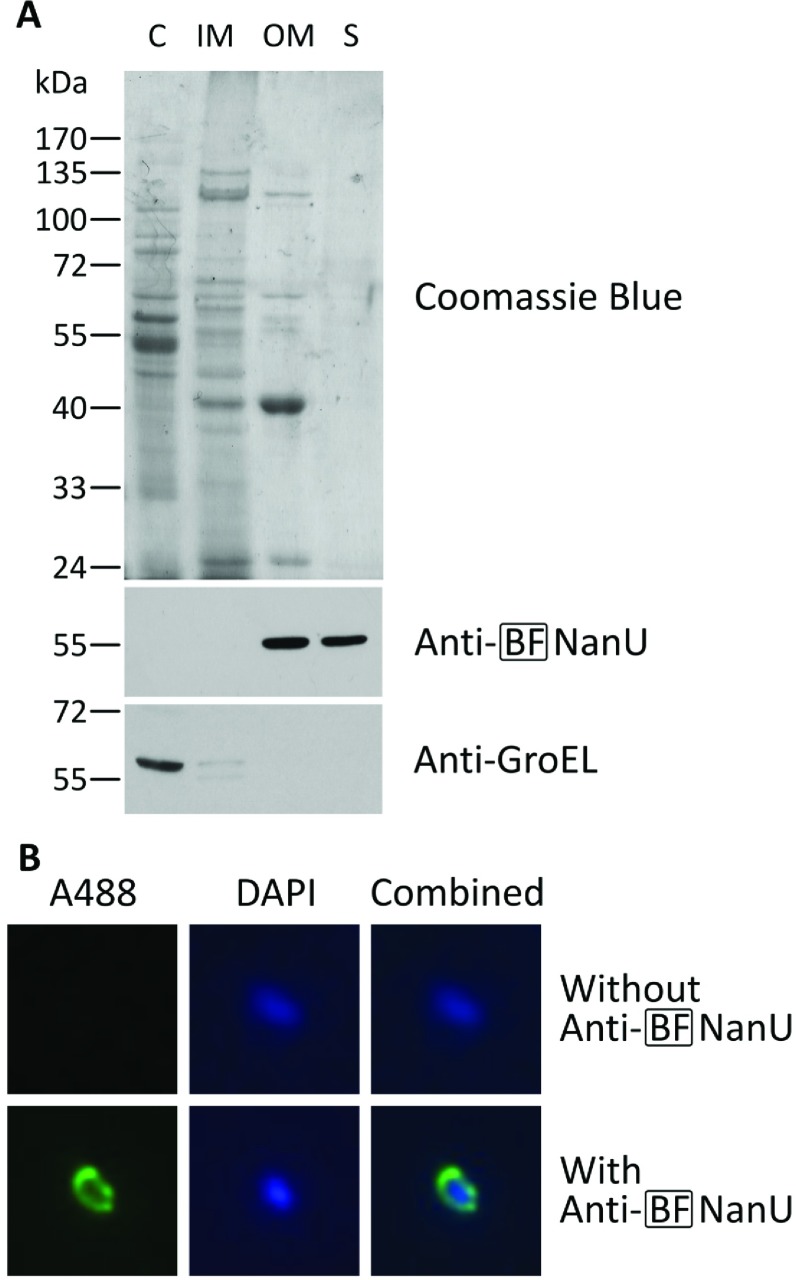
Cellular localization of NanU in *B. fragilis* (**A**) Cell fractions isolated from *B. fragilis* NCTC 9343 cells were obtained by differential detergent fractionation as described in the Materials and methods section. Protein (10 μg) from the cytoplasmic (C), inner membrane (IM), outer membrane (OM) and 20× concentrated secreted (S) fractions were loaded into each lane, resolved by SDS/PAGE (10% gel) and stained with Coomassie Blue. Parallel gels were blotted on to nitrocellulose membranes and probed with BF-NanU rat antiserum and rabbit anti-(*E. coli* GroEL) as described above. The blots were incubated with HRP-conjugated anti-(rat goat IgG) or anti-(rabbit goat IgG) and visualized. Molecular mass is given on the left-hand side in kDa. (**B**) Representative micrographs of immobilized intact *B. fragilis* cells, probed with BF-NanU rat antiserum (lower row) or just PBS (upper row) and incubated in the dark with goat anti-(rat IgG–Alexa Fluor™ 488) (A488) and counterstaining with DAPI. Fluorescence was visualized at ×100 magnification using appropriate filters, with NanU (left-hand panels) and DNA (middle panels) shown in green and blue respectively, alongside a combined image (right-hand panels).

Both bioinformatic predictions (presence of Sec-dependent signal sequence and similarity to SusCD protein systems) and functional studies, i.e. dependency on TonB, indicated that the NanOU proteins might be associated with the outer membrane in both *T. forsythia* and *B. fragilis* [18,30]. To test the localization of NanU, we performed a differential detergent-based cell fractionation and examined the fractions by SDS/PAGE and Western blotting using a BF-NanU antiserum, which we generated from purified His_6_–NanU from *B. fragilis*. These data clearly show that NanU is associated with the outer membrane of *B. fragilis*, but is not found in the cytoplasmic or inner membrane fractions (Figure 4A). In addition, we performed a control using a commercially available anti-GroEL antibody, which was raised against the *E. coli* GroEL protein, but which has 76% primary amino acid sequence similarity with GroEL from *B. fragilis* and reacts with a 60 kDa protein found only in the cytoplasm of the intracellular fractions (Figure 4A).

We also determined, via TCA precipitation of (20× concentrated) liquid culture supernatants of *B. fragilis*, that small quantities of NanU were present in these fractions, whereas we could find no trace of the intracellular marker GroEL. This indicates that a small amount of NanU, probably in a ratio of at least 20:1, may also be released from the surface into the surro-unding medium. To further characterize and examine the surface localization of the NanU protein, we examined its cellular localization *in situ* by the use of immunofluorescence microscopy by probing immobilized intact *B. fragilis* cells with our anti-NanU antibody. As illustrated in Figure 4(B), the anti-NanU antibody highlighted an immunoreactive protein on the surface periphery of *B. fragilis*, indicating NanU localization on the cell surface.

### BF-NanU and TF-NanU are monomeric high-affinity sialic acid-binding proteins

**Figure 5:**
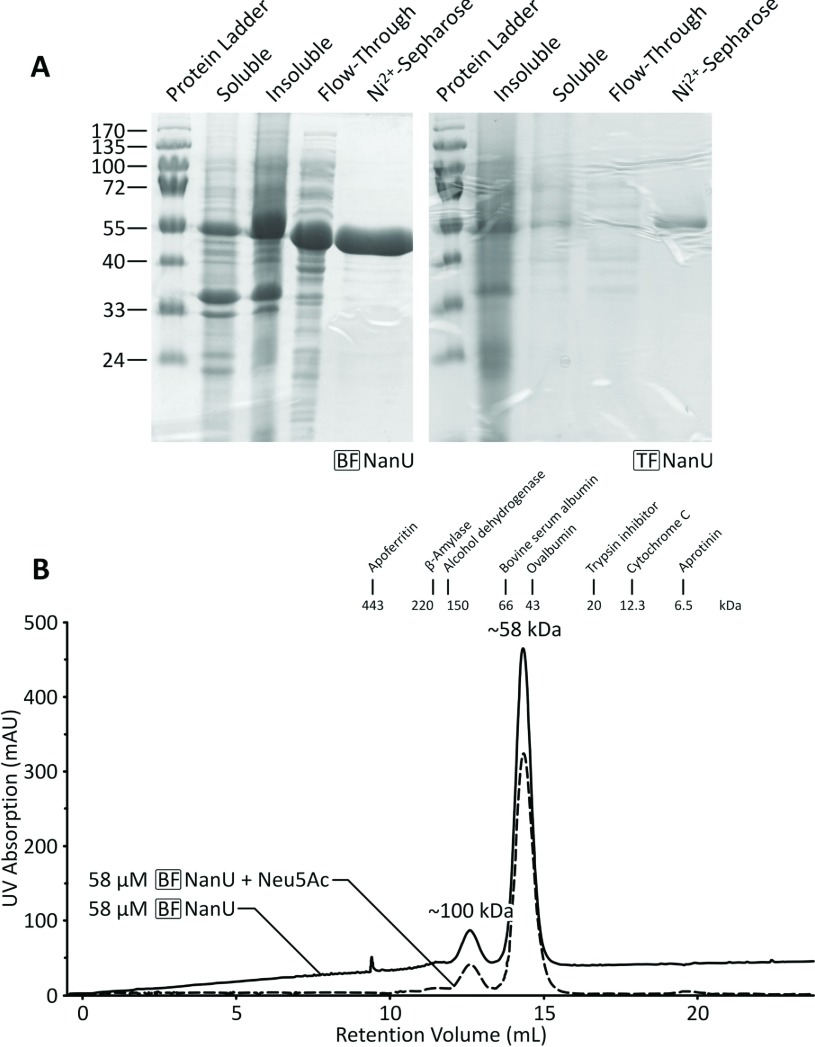
Affinity-tag purification of BF-NanU and TF-NanU proteins (**A**) PCR-amplified C-terminally His_6_-tagged versions of BF-NanU (*BF1720*) and TF-NanU (*TF0034*) were expressed in *E. coli* BL21λ(DE3) cells containing relevant pET21a(+) derivatives and purified (see the Materials and methods section) before analysis by SDS/PAGE. Molecular mass is given on the left-hand side in kDa. (**B**) Purified BF-NanU (3 mg/ml), with and without pre-incubated Neu5Ac at an equimolar concentration, were sequentially applied to a HiLoad Superdex 200 PG gel-filtration column, calibrated with a gel-filtration standard of which the protein peaks and their corresponding elution volumes are represented by vertical lines. Both NanU and NanU–Neu5Ac migrated as globular species with an apparent molecular mass of ~58 kDa protein, approximating that of a monomer (57 kDa). mAU, milli-absorption unit.

Given its homology with SusD and having established its outer membrane localization, we then tested our hypothesis that NanU is a high-affinity sialic acid-binding protein. To achieve this, we expressed native *B. fragilis* and a codon-optimized copy of the *T. forsythia nanU* genes in pET21a(+) with a C-terminal His_6_ tag. Both of these 57 kDa proteins were expressed at high levels in *E. coli* BL21λ(DE3) cells and purified to homogeneity (Figure 5A). Both the BF-NanU and TF-NanU proteins isolated were found to be largely monomeric both in the presence and absence of free sialic acid by size-exclusion chromatography (Figure 5B).

**Figure 6:**
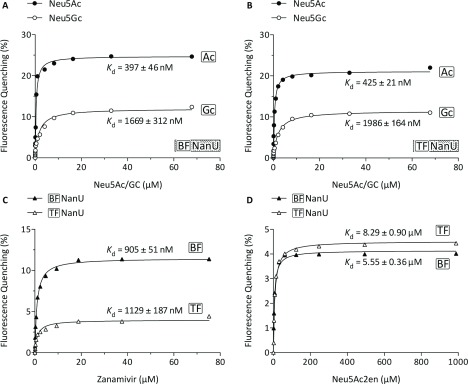
Steady-state tryptophan fluorescence titration experiments to probe sialyl-sugar-binding affinities of NanU Quenching of intrinsic tryptophan fluorescence was measured (λ_ex_=295 nm and λ_em_=330 nm) at 25°C when different concentrations of Neu5Ac or Neu5Gc (**A** and **B**), zanamivir (**C**) and Neu5Ac2en (**D**) were added to 0.5 μM NanU. The fluorescence change was normalized to account for dilution effects and the data shown are representative of three independent experiments. Equilibrium dissociation constants (*K*_d_) were determined using an one-site specific binding equation.

**Figure 7:**
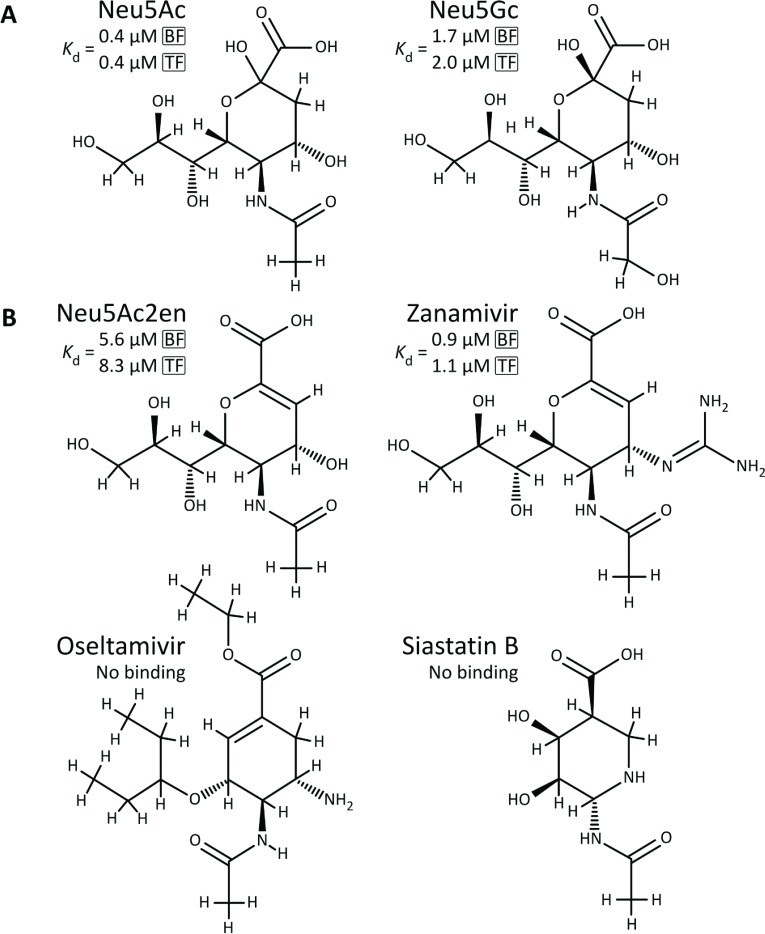
Schematic representation of Neu5Ac, Neu5Gc (A) and various sialidase inhibitors (B) orientated to illustrate the differences in their R groups around the 5-carbon furanose ring

The ability of these proteins to bind sialic acid was assessed by steady-state tryptophan fluorescence and *K*_d_ values were derived according to the curves fitted to the titration data. These data clearly show that the NanU proteins from *B. fragilis* and *T. forsythia* both bind sialic acid (Neu5Ac) with a very high affinity, displaying *K*_d_ values of 397 and 425 nM respectively (Figure 6A, ●). As these proteins have 86.5% similarity and 63% identity at the amino acid level and both complement our *E. coli* transport-deficient strain equally, such similarity in *K*_d_ value might have been expected. Although Neu5Ac is the most common form of sialic acid present in the human body, there are other forms of sialic acid to which both *B. fragilis* and *T. forsythia* are exposed in the human gut or oral cavity respectively. One significant example of this is Neu5Gc (*N*-glycolylneuraminic acid), which differs from Neu5Ac by the presence of an extra oxygen atom at position C_5_ of the sugar (Figure 7A). Despite this small difference, hydroxylated Neu5Gc is recognized by the human body as a ‘foreign’ antigen and indeed Neu5Gc is produced and presented on the surface of cells in higher (non-human) mammals [31]. However, although it is not synthesized *de novo* by human cells, it can be incorporated into human glycoproteins if supplied exogenously, usually from dietary sources [32]. In this context, we tested the ability of NanU from both *T. forsythia* and *B. fragilis* to bind Neu5Gc *in vitro* and showed that it still binds with high affinity, 1.7 and 2 μM respectively (Figures 6A and 6B, ○), although these *K*_d_ values were significantly higher than for Neu5Ac (Figures 6A and 6B, ●). In addition, we also tested the binding ability of the sialic acid analogue zanamivir and the closely related compound Neu5Ac2en (2-deoxy-2,3-didehydro-Neu5Ac). Surprisingly, zanamivir binds NanU with a higher affinity than the semi-native ligand Neu5Gc with *K*_d_ values of 0.9 and 1.1 μM with the *B. fragilis* and *T. forsythia* proteins respectively (Figure 6C). However, Neu5Ac2en bound with significantly higher *K*_d_ values of 5.6 (BF-NanU) and 8.3 μM (TF-NanU) (Figure 6D). In contrast, neither of the chemically related sialidase inhibitors oseltamivir or siastatin B, with a similar structure to Neu5Ac as illustrated in Figure 7, exhibited significant binding in our experiments. In addition, the model sialo-conjugate sugars 3′- and 6′-siallyl-lactose, which we have shown previously as targets for the NanH sialidase from *T. forsythia* [33], did not display any interaction with NanU, indicating that this protein only interacts with free sialic acid (or analogues) and not directly with sialylated glycoprotein-derived conjugates. To eliminate the possibility of contamination by adventitious ligand, the BF-NanU protein was also overexpressed in the same *E. coli* strain, but with growth on M9 minimal medium with 15 mM glycerol at 25°C overnight to eliminate adventitiously bound ligand affecting the steady-state binding kinetics. The BF-NanU expressed and purified from the cells grown in M9 medium plus glycerol also exhibited a similar tight binding with Neu5Ac with a *K*_d_ value of 409 nM (results not shown), confirming that our purification protocols and the extensive dialysis of NanU proteins expressed in LB medium were suitable for producing unbound proteins.

### The crystal structure of BF-NanU reveals a novel binding pocket structure

**Figure 8:**
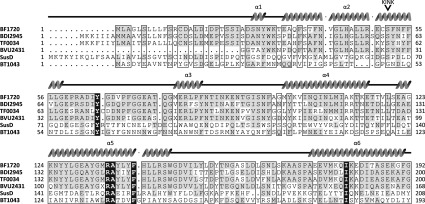
Sequence alignment of NanU with SusD family members The sequence alignment profile of the N-terminal region was generated using the Multalin Server (http://multalin.toulouse.inra.fr/multalin). Structural motifs predicted for BF1720 are presented on the top of the sequence alignment, where spirals represent α-helices. The kink in the α2 helix is indicated by an arrowhead. Conserved residues across the orthologues are highlighted in grey. The sources of these SusD proteins are as follows: BF1720 from *B. fragilis* NCTC 9343, BDI2495 from *Parabacteroides distasonis* A.T.C.C. 8503, TF0034 from *T. forsythia* A.T.C.C. 43037, BVU2431 from *B. vulgatus* A.T.C.C. 8482, and SusD from BT1043 from *B. thetaiotaomicron* VPI-5482 (PDB code 3CKC) and *B. thetaiotaomicron* VPI-5482 (PDB code 3EHN).

**Figure 9:**
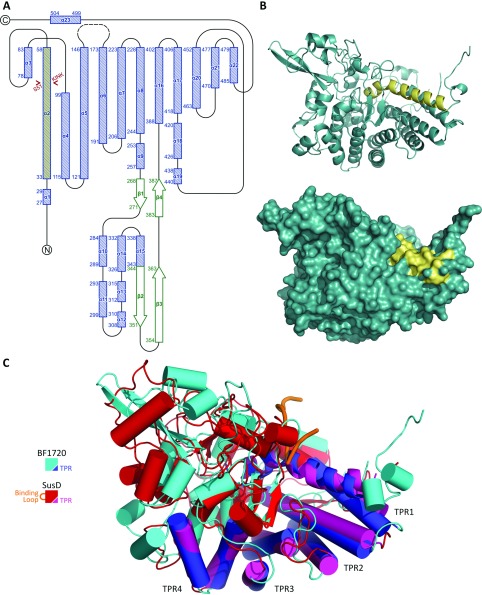
The 3D structure of BF-NanU X-ray diffracting crystals of BF-NanU were grown in 0.2 M ammonium acetate, 20% (w/v) PEG 3350 and data were collected to a final resolution of 1.6 Å. (**A**) Topology diagram of BF-NanU. Random coils, α-helices and β-strands are shown as continuous lines, shaded bars and open arrows respectively, with residue numbers marking the extent of the secondary structure elements. α2, is highlighted in yellow. The missing electron density corresponding to the loop region that links α5 with α6 is represented by a broken line. (**B**) Cartoon and space-filling representations of BF-NanU with α2 highlighted in yellow. (**C**) Topology-based alignment of BF-NanU (BF1720; PDB code 4L7T; coloured cyan with TPRs in blue) with chain A of the apo SusD from *B. thetaiotaomicron* VPI-5482 (PDB code 3CKC; coloured red with TPRs in magenta and the maltoheptose-binding loop in orange). The co-ordinates were superimposed in Coot with the SSM (secondary structure matching) algorithm. The RMSD of the C_α_ atoms of the superimposed molecules based upon 255 residues in the TPR motifs was 1.56 Å.

To characterize further the NanU protein, we determined the crystal structure of BF-NanU to a resolution of 1.6 Å. The crystal asymmetric unit shows clearly a monomeric form for the protein in agreement with our gel-filtration data (Figure 5B), and an overall predominantly α-helical structure, reminiscent of several other members of the SusD family (Figure 8). In keeping with other members of the SusD family and despite low primary sequence similarity (~20%), virtually all the secondary structure elements are maintained between BF-NanU and the founder member of this group, the *B. thetaiotaomicron* SusD starch-binding protein, both in the apo form (PDB code 3CKC) and in complex with maltoheptaose (PDB code 3CK9). These features include the four TPR (tetratricopeptide repeat) units formed from the α-helical pairs TPR1, α2 (residues 33–58) with α4 (residues 99–115), TPR2, α5 (residues 121–146) with α6 (residues 173–191), TPR3, α7 (residues 206–223) with α8 (residues 228–244) and TPR4, α16 (residues 388–402) with α17 (residues 406–418), which are postulated to be involved in interaction with their cognate TBDRs and also form a scaffold for the rest of the SusD protein structure [34]. With the exception of an extended β-ribbon and turn (Met^344^–Cys^363^) in BF-NanU and two small two-stranded anti-parallel β-sheets in SusD (Cys^94^–Trp^96^ and Ile^323^–Ala^325^ and Lys^172^–Phe^175^ and Val^185^–Lys^188^), the majority of structural differences lie within the loop regions (Figure 9A). Most notably, in SusD, there is a loop region inserted between the first two helices (residues 70–77 in SusD; Figure 9C, orange loop), much of which only becomes ordered upon binding to the non-physiological ligand maltoheptaose, where it makes important contacts and is likely to be important for starch binding by SusD [34]. There is no equivalent loop in BF-NanU; instead, this displays an extended helix that is kinked by ~35° between Cys^50^ and Ser^51^ and extends to Gly^58^ (α2 in Figure 9A, and highlighted yellow in Figure 9B). This difference in the structures between SusD and NanU is highlighted by a structural alignment (Figure 9C), with the extended kinked helix of NanU highlighted in blue and the corresponding region of the apo-form of SusD in magenta, where two short helices with a different orientation are separated by a short linker (orange). The potential importance of this helix is highlighted further by an amino acid sequence alignment with three other putative SusD family NanU homologues from *T. forsythia* (TF0034), *Parabacteroides distastonis* (BDI_2945) and *B. vulgatus* (BVU_2431) with the SusD and BT1043 proteins from *B. thetaiotaomicron* (Figure 8). The α2 helix defines a motif pivoted around amino acid 50/51 (extending from Glu^33^ to Gly^58^) that is well conserved between the putative NanU proteins (Figure 8, residues highlighted in grey text), but not with non-sialic acid-related Sus proteins, namely SusD and BT1043 from *B. thetaiotaomicron*, both which are involved in oligosaccharide rather than monosaccharide binding [34,35].

A second structural loop in SusD, seen to move upon substrate binding (residues 292–297), and thus provide a critical tyrosine-mediated contact, is present in BF-NanU (residues 276–280), but there are no structurally equivalent residues to the tyrosine and tryptophan residues that co-ordinate ligand in the SusD co-crystals. At present, we can only speculate as to the binding site for sialic acid, as we have tried repeatedly to produce ligand-bound crystals using various ligands with no success to date, but it seems possible that the observed differences in structure and amino acid composition may define the binding specificity of BF-NanU for monosaccharide derivatives, such as sialic acid, over the starch or oligomeric sugars observed in SusD family protein structures to date [34,35].

## DISCUSSION

In the present study, we establish that the NanOU sialic acid-transport system plays a role in sialic acid transport in *B. fragilis*. The role of sialic acid in the lifestyle of human-associated commensal and pathogenic bacteria is, at least partly, dependent on their ability to scavenge and transport sialic acid across both membranes of these Gram-negative organisms [10,14]. The results of the present study suggests that the NanOU system is functional in at least two members of the Bacteroidetes, playing a role not only in the sialic acid-rich environment of the oral cavity, but also in the human gastrointestinal tract [8]. We also show that the *nanOU* function is dependent on the presence of an intact TonB–ExbB–ExbD inner membrane energizing complex since activity was lost on a Δ*tonB* background. One assumes therefore that NanO from *B. fragilis* and *T*. *forsythia* can interact with TonB from *E. coli*, possibly suggesting a conserved mode of recognition across at least these two distantly related genera, presumably dependent on the conserved TonB-box motifs within these proteins. Furthermore, the present study represents the first example of a TonB-dependent transporter module adapted for sialic acid and builds upon growing evidence that TonB-dependent transporters are employed for sugar transport [36,37].

Specifically, we have established NanU as a novel member of the SusD family of proteins that binds sialic acid with a high affinity (*K*_d_ of ~400 nM). In contrast, the only binding data regarding SusD family proteins characterize interactions with non-physiological ligands such as cyclodextrin by SusD (in the hundreds of μM range) [34] and *N*-acetyl lactosamine by BT1043 in the mM range [35], i.e. NanU binds sialic acid several orders of magnitude more avidly than any member of this family studied to date and is the first characterized with its native ligand. The present study also reveals that NanU binds less efficiently to the related, but non-human-derived, sugar Neu5Gc with an affinity 5-fold lower than Neu5Ac. This might provide biochemical evidence for our observations in biofilm studies that *T. forsythia* utilizes Neu5Gc less efficiently than Neu5Ac [18]. The high affinity we observe of NanU for Neu5Ac is significantly higher than that of the lectin domain of the *Vibrio cholerae* sialidase (30 μM) [38] and may reflect its role in transport compared with the *Vibrio* sialidase which has a dual role in receptor unveiling and nutrition. In agreement with this idea, the periplasmic sialic-binding protein SiaP, which binds sialic acid as part of a high-affinity tripartite ATP-independent periplasmic transporter in *H. influenzae*, has an even higher affinity with a *K*_d_ value for sialic acid of 120 nM [39]. The binding of NanU by zanamivir is a tantalizing observation given that this was designed as a sialic acid transition state analogue aimed at inhibiting viral sialidase enzymes. This, therefore, opens up the possibility that sialidase inhibitors (or more precisely sialic acid analogues) have the potential as dual-action inhibitors of sialic acid utilization systems as they may act both on sialidases [18] and transport systems.

Therefore NanU, together with its partner porin NanO, forms part of an integrated sialic acid-uptake system present in several Bacteroidetes species which we suggest as the first sialic acid-specific PUL. In this context, the most well-studied TonB-dependent PUL in bacteria is the SusCD starch processing and uptake system of *B. thetaiotaomicron* [20]. In the SusCD system, SusD is the direct analogue of NanU, acting as an outer membrane-associated protein that binds starch polymers and holds them in place for the glycosyl hydrolase SusG to act upon before transport of smaller oligosaccharides across the outer membrane by SusC. In contrast, the results of the present study suggest that NanU does not bind glycoconjugated forms of sialic acid (e.g. sialyl-lactose), that are present as the terminal moieties of many human glycoproteins and gangliosides. Rather, it binds the monosaccharide sialic acid, probably after it has been released from glycoproteins by the action of NanH sialidase. Although we have no information at this stage on the mechanism of release of sialic acid from NanU, we postulate that interaction with NanO may precipitate a conformational change in NanU that acts to release sialic acid to NanO. In addition, although our data show that NanU is surface localized, several questions remain that we are investigating. The first being that since NanU does not possess a lipid anchor, how does it become localized to the outer membrane? The most simple answer is that this is dependent on interaction with NanO. The second surrounds how it is secreted to the surface of the cell. NanU possesses a typical type II signal-recognition particle signal for passage across the inner membrane, but how it traverses the outer membrane, as other SusD family proteins, is currently not known. It is of note that NanU does not contain a CTD (C-terminal domain) motif for type IX secretion that is a feature of Bacteroidetes species. We also observed that a proportion of NanU is found in the cell-free fraction, indicating either its secretion from the cell surface or, possibly, an association with OMVs (outer membrane vesicles) that *B. fragilis* is well known to release and with which various glycosidase activities, including sialidase, have been associated [40].

The results of the present study also suggest that *nanO* alone is able to almost completely restore sialic acid-dependent growth in *E. coli* devoid of sialic acid outer membrane permeases. Therefore NanU must either improve the efficiency of this system in the gut and oral cavity, where both competition for sialic acid and its bioavailability might be reduced, or that NanU acts to store sialic acid on the surface of these bacteria as has been suggested for the starch-binding protein SusD [40]. Another possibility is that NanU may act to receive sialic acid directly from the secreted NanH sialidase, which is known to be key to growth on sialoglycoproteins in *T. forsythia* [33].

We also report the structure of NanU at 1.6 Å and reveal that despite low sequence similarity with other SusD proteins (~20% at the amino acid level), there is a strongly conserved structural homology based mainly on the presence of a series of four α-helix-rich TPR units that define the scaffold for the structure of these proteins [34,35]. It is these TPRs that are postulated to form complimentary interacting surfaces for their TBDR partners, but we also posit that this may mediate interaction with the cognate sialidase NanH as well. Although the SusD scaffold is structurally well conserved, the binding pockets for saccharide ligands exhibit a larger degree of plasticity [34,35]. This seems to be further borne out by the structure of NanU, which we report in the present paper has an extended kinked helix in addition to several other structural differences that probably reflect its preference for a negatively charged monosaccharide ligand.

Finally, we observed sialic acid-responsive expression of NanU in *B. fragilis*. This might be expected given that a previous study in *B. fragilis* illustrated that deletion of the *nanR* gene, encoding a putative sialic acid-responsive helix–turn–helix-containing transcriptional repressor located 5′ of the *nan* operon (*BF1806* -*1809*; Supplementary Figure S1 at http://www.biochemj.org/bj/458/bj4580499add.htm) led to constitutive expression of sialic acid aldolase activity (encoded by the *nanL* gene) and that aldolase activity increased in response to elevated sialic acid levels. The results of the present study, alongside other published work [8,41], illustrate that expression of catabolic (*nanLE*), transport (*nanT* and *nanOU*) and genes encoding a sialidase (*nanH*), hexosaminidase (*nahA*) and a putative sialate-*O*-acetylesterase (*estS*) increases in the presence of sialic acid. However, the mechanistic details of this potential *nanR*-mediated sialic acid-dependent derepression remain unclear.

In summary, we have presented evidence that NanU proteins are prototype members of a new class of high-affinity sialic acid-binding proteins of the SusD family. We also establish their dependence on TonB and thus highlight further the important role of TonB-dependent transporters as part of PUL in gut- and oral-dwelling Bacteroidetes. There, however, remain several questions regarding the series of protein–protein interactions in this system, namely with the sialidase and outer membrane porin, and, furthermore, the sialic acid-binding characteristics of the NanO porin. In addition, it remains to be elucidated the exact mechanism of co-ordination of sialic acid by the NanU protein, which is clearly high affinity, but yet seems to function via the use of an unusual motif. These and further questions are currently under investigation in our group.

## Online data

Supplementary data
