# Supplementary material for: Structural and functional characterization of NanU, a novel high-affinity sialic acid-inducible binding protein of oral and gut-dwelling Bacteroidetes species
Source: Biochem J. 2014 Feb 28;458(Pt 3):499–511. doi: 10.1042/BJ20131415 (PMC3969230; doi:10.1042/BJ20131415)
Supplement: Supplementary data [file bj4580499add.pdf]

Biochem. J. (2014) **458**, 499–511 (Printed in Great Britain) doi:10.1042/BJ20131415

SUPPLEMENTARY ONLINE DATA

Structural and functional characterization of NanU, a novel high-affinity sialic acid-inducible binding protein of oral and gut-dwelling Bacteroidetes species

Chatchawal PHANSOPA*, Sumita ROY*, John B. RAFFERTY†, C. W. Ian DOUGLAS*, Jagroop PANDHAL‡, Phillip C. WRIGHT‡, David J. KELLY† and Graham P. STAFFORD*[1]

*School of Clinical Dentistry, University of Sheffield, Sheffield S10 2TA, U.K.
†Department of Molecular Biology and Biotechnology, University of Sheffield, Sheffield S10 2TN, U.K.
‡Department of Chemical and Biological Engineering, University of Sheffield, Sheffield S1 3JD, U.K.

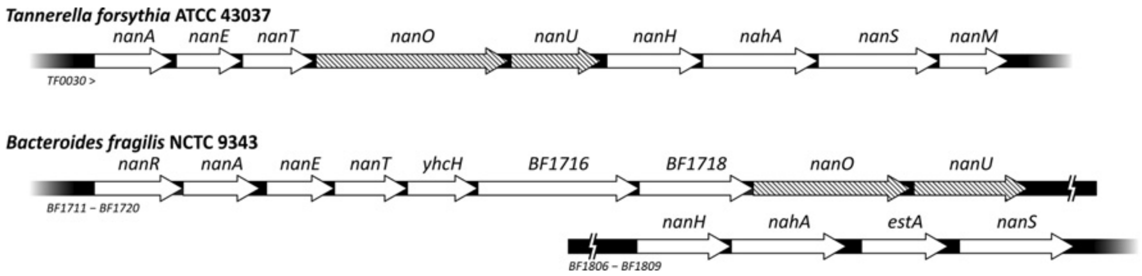

Figure S1 Sialic acid catabolism and transport clusters in *T. forsythia* and *B. fragilis*

Predicted and confirmed sialic acid utilization genes and their orientations are illustrated using standard *nan* gene descriptors based on the genome sequences of the organisms. *nanOU* are shaded, whereas the *nan* genes of *B. fragilis* are organized in discontinuous clusters.

Table S1 Strains and plasmids used in the present study

Strain/plasmid	Relevant characteristics	Source/reference
Strains		
E. coli MG1655	F^- λ^- *ilvG*$^-$ *rfb*-50 *rph*-1	Professor Barry Wanner, Purdue University, West Lafayette, IN, U.S.A.
E. coli Δ*tonB*	MG1655 Δ*tonB*::*FRT-Km-FRT*	The present study
E. coli Δ*nanCnanR*Δ*ompR*	MG1655 Δ*nanCnanR*(*amber*) Δ*ompR*::*Tn10*(*tet*)	[1]
E. coli Δ*nanCnanR*Δ*ompR*	MG1655 Δ*nanCnanR*(*amber*) Δ*ompR*::*Tn10*(*tet*) Δ*tonB*(*FRT-Km-FRT*)	The present study
E. coli BL21λ(DE3)	F^- *ompT gal dcm lon hsdS*$_B$(r_B^- m_B^-) λ(DE3 [*lacI lacUV5-T7 gene 1 ind1 sam7 nin5*])	New England Biolabs
E. coli DH5α	*fhuA2*Δ(*argF-lacZ*)*U169 phoA glnV44* φ*80* Δ(*lacZ*)*M15 gyrA96 recA1 relA1 endA1 thi*$^-$ *1 hsdR17*	New England Biolabs
T. forsythia NCTC 43037	Wild-type	Professor William Wade, King's College London, London, U.K.
B. fragilis NCTC 9343	Wild-type	Professor Sheila Patrick, Queen's University Belfast, Belfast, U.K.
Plasmids		
pBAD18		[2]
pCP-cBFO	*BF1719* (BF-*nanO*) cloned into pBAD18	The present study
pCP-cBFU	*BF1720* (BF-*nanU*) cloned into pBAD18	The present study
pCP-cBFOU	*BF1719-BF1720* (BF-*nanOU*) cloned into pBAD18	The present study
pCP-cTFOU	*TF0033-TF0034* (TF-*nanOU*) cloned into pBAD18	The present study
pCP-eBFU	BF-*nanU*$_{His}$ cloned into pET21a(+)	The present study
pCP-eTFU	TF-*nanU*$_{His}$ cloned into pET21a(+)	The present study

[1] To whom correspondence should be addressed (email g.stafford@sheffield.ac.uk).
Structural factors and co-ordinates for the *B. fragilis* NanU have been deposited in the PDB under code 4L7T.

© 2014 The Author(s)
The author(s) has paid for this article to be freely available under the terms of the Creative Commons Attribution Licence (CC-BY) (http://creativecommons.org/licenses/by/3.0/) which permits unrestricted use, distribution and reproduction in any medium, provided the original work is properly cited.

Biochemical Journal www.biochemj.org

Table S2 Oligonucleotide primers used in the present study

Restriction endonuclease sites are underlined and FRT (Flp recombinase target) sites are highlighted in bold.

Primer	Sequence
BF*nanO*-XbaI-F	5′-AATATCTAGAAATAATTTTGTTTAACTTTAAGAAGGAGATATAC ATATGAAGAAAACCATCTTCTTG-3′
BF*nanO*-HindIII-R	5′-AATAAAGCTTTCAGAATGTCACTGACAA-3′
BF*nanU*-XbaI-F	5′-AATATCTAGAAATAATTTTGTTTAACTTTAAGAAGGAGATATAC CATGTTAGCAGGGCTT-3′
BF*nanU*-SphI-R	5′-AATAGCATGCTCAATTCTGATATCCCGGAG-3′
TF*nanO*-XbaI-F	5′-AATATCTAGATAATTTTGTTTAACTTTAAGAAGGAGATATACATA TGAAAGGAATTTTAAAAAAAT-3′
TF*nanU*-SphI-R	5′-AATATCTAGATTATTCATACCCCGGAGT-3′
EC*tonB*-FRT-F	5′-**TTGCATTTAAA**ATCGAGACCTGGTTTTTCTACTGAATCAGTGTA GGCTGGAGCTGCTTC-3′
EC*tonB*-FRT-R	5′-CCTGTTGAGTAATAGTCAAAAGCCTCCGGTCGGAGTGCATTCCG **GGGATCCGTCGACC**-3′
BF*nanU*-NdeI-F	5′-AATACATATGCTGGATATTGATCCT-3′
BF*nanU*-XhoI-R	5′-AATACTCGAGATTCTGATATCCCGG-3′

Table S3 Data collection and refinement statistics for BF-NanU

Values in parentheses correspond to the highest-resolution shell. R_{pim} (precision-indicating merging *R* factor) was calculated using the formula $R_{pim} = \Sigma hkl[1/N-1)]^{1/2}\Sigma_i|I_i(hkl) - I(hkl)|/\Sigma hkl\Sigma_i I_i(hkl)$. *R* factor $= \Sigma|F_{obs} - F_{calc}|/\Sigma F_{obs}$. R_{free} was calculated as for the *R* factor, but using 5 % of experimental data excluded from refinement for validation.

Parameter	Value
Data collection	
Space group	$P2_12_12_1$
Unit cell (Å)	$a = 65.5$ Å, $b = 88.5$ Å, $c = 99.1$ Å, $\alpha = \beta = \gamma = 90°$
Resolution range (Å)	52.67–1.61 (1.65–1.61)
Number of measured reflections	499575 (36868)
Number of unique reflections	75284 (5480)
Completeness (%)	99.9 (99.9)
R_{pim}	0.045 (0.468)
Mn (S.D.)	14.5 (2.6)
Refinement	
R/R_{free}	19.1/23.2
RMSD in bond distances (Å)	0.026
RMSD in bond angles (°)	2.04
Ramachandran	
Most favoured (%)	95.5
Additionally allowed (%)	4.3

REFERENCES

1 Roy, S., Douglas, C. W. I. and Stafford, G. P. (2010) A novel sialic acid utilization and uptake system in the periodontal pathogen *Tannerella forsythia*. J. Bacteriol. **192**, 2285–2293

2 Guzman, L. M., Belin, D., Carson, M. J. and Beckwith, J. (1995) Tight regulation, modulation, and high-level expression by vectors containing the arabinose PBAD promoter. J. Bacteriol. **177**, 4121–4130

Received 31 October 2013/11 December 2013; accepted 19 December 2013
Published as BJ Immediate Publication 19 December 2013, doi:10.1042/BJ20131415

© 2014 The Author(s)
The author(s) has paid for this article to be freely available under the terms of the Creative Commons Attribution Licence (CC-BY) (http://creativecommons.org/licenses/by/3.0/) which permits unrestricted use, distribution and reproduction in any medium, provided the original work is properly cited.
